# Gastrointestinal Pyogenic Granuloma (Lobular Capillary Hemangioma): An Underrecognized Entity Causing Iron Deficiency Anemia

**DOI:** 10.1155/2016/4398401

**Published:** 2016-06-15

**Authors:** Marshall W. Meeks, Umar M. Kamal, Muhammad B. Hammami, Jason R. Taylor, M. Louay Omran, Yongxin Chen, Jin-Ping Lai

**Affiliations:** ^1^Department of Pathology, Saint Louis University School of Medicine, Saint Louis, MO 63104, USA; ^2^Department of Gastroenterology and Hepatology, Saint Louis University School of Medicine, Saint Louis, MO 63104, USA

## Abstract

Pyogenic granuloma (PG), more accurately known as lobular capillary hemangioma, is a benign vascular tumor that usually occurs in the skin or oral mucosa. This lesion is rarely reported in the gastrointestinal tract but is known to bleed if not resected. We herein describe a case series with the clinical, endoscopic, and histologic findings of four cases of gastrointestinal PG at our institution. In addition, we provide a review of the literature and summation of all reported cases of PG specific to the gastrointestinal tract. Based on our experience, we suggest that the actual incidence of gastrointestinal PG may in fact be higher than reported because PG can be unrecognized or improperly diagnosed. It is important for the clinician to properly recognize this lesion as a source of anemia and its propensity to bleed during biopsy or resection.

## 1. Introduction

Pyogenic granuloma (PG), or more accurately known as lobular capillary hemangioma, is a benign vascular tumor characterized as bright red papules with a friable surface that may bleed profusely from minor trauma. PG typically affects the skin and oral mucosa, but a few cases have been reported in the gastrointestinal (GI) tract [1]. Given this lesion's vascular structure, severe anemia can develop if not recognized and correctly treated [2].

In this case series, we report four new cases of PG in the GI tract. Two patients with gastric PGs presented with melena and symptomatic anemia and were successfully treated with endoscopic polypectomy. A third patient presented with a PG with no stigmata of recent bleeding. The last patient was incidentally found to have a PG at the site of perforated Meckel's diverticulum after a small bowel resection. Here, we discuss the endoscopic and pathologic findings of these cases in addition to a review of the literature.

## 2. Case Reports

**Figure 1:**
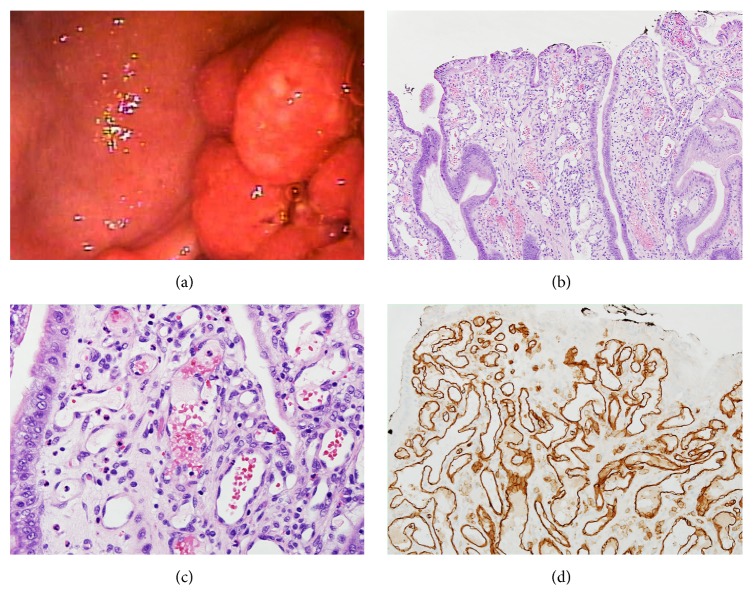
Pyogenic granuloma of the stomach (Case 1). (a) Endoscopic appearance of the 40 mm gastric polypoid nodule with surface erosion. (b-c) Histologically, a capillary hemangioma is present and arranged in a lobular pattern and filled with clusters of small capillary vessels ((b), ×100; (c), ×400). (d) Immunohistochemistry of CD31 highlighting the capillary proliferation (×400).

*Case* *1*. A 70-year-old Caucasian female with a past medical history significant for end-stage renal disease, ulcerative colitis (maintained on mesalamine), and cirrhosis complicated by portal hypertensive gastropathy presented to our institution for elective endoscopic resection of a gastric polyp. She presented to an outside hospital two months priorly with complaints of intermittent bloody stools, up to eight stools per day, as well as early satiety and fatigue. Her hemoglobin was 8.1 g/dL (down from 9.6 g/dL a month earlier), for which she received a transfusion. Outside hospital esophagogastroduodenoscopy (EGD) revealed a 40-millimeter gastric polyp (Figure 1(a)). Endoscopic mucosal resection was attempted but aborted due to bleeding. At our institution, the polyp was partially resected with a piecemeal technique using a hot snare after injection of epinephrine. Eight hemostatic clips were successfully placed over the polypectomy site to prevent further bleeding. Histology from the polyp revealed inflammatory and hyperplastic changes with features of lobulated capillary hemangioma and granulation tissue (Figures 1(b)–1(d)).

**Figure 2:**
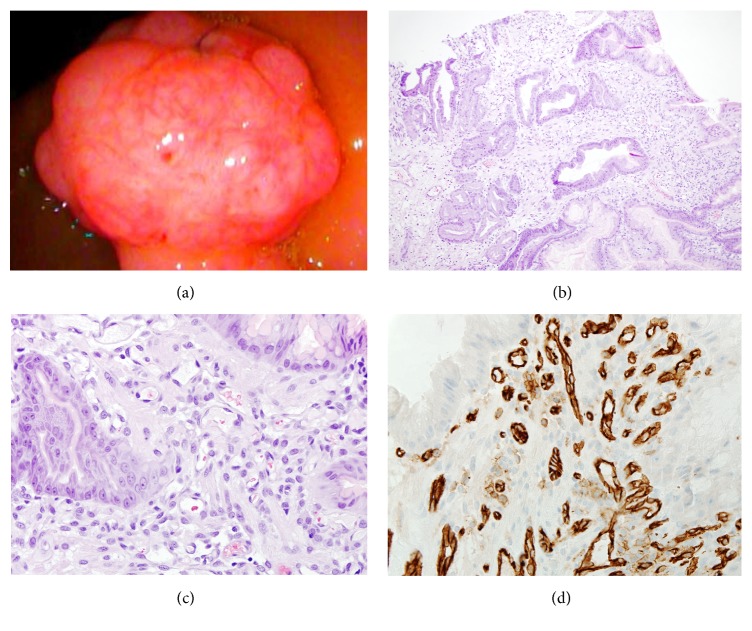
Pyogenic granuloma of the stomach (Case 2). (a) Endoscopic appearance of the 12 mm gastric polypoid nodule with surface erosions. (b-c) Histologically, a capillary hemangioma is present and arranged in a lobular pattern and filled with clusters of small capillary vessels and neutrophils infiltrate ((b), ×100; (c), ×400). (d) Immunohistochemistry of CD31 highlighting the capillary proliferation (×400).

*Case* *2*. A 70-year-old Caucasian male was admitted for melena in the setting of dual antiplatelet therapy (DAPT). The patient had a medical history significant for end-stage renal disease secondary to chronic hypertension and coronary artery disease. He reported three black-tarry stools daily, starting eight days prior to admission. Coagulation profile was normal and presenting hemoglobin was 7.8 g/dL. EGD revealed a twelve-millimeter, oozing, and pedunculated polyp in the distal antrum with migration into the duodenal bulb (Figure 2(a)). Due to the DAPT, the polyp was only biopsied. In a subsequent endoscopy, the polyp was completely resected with a hot snare after injection with epinephrine. Three hemostatic clips were placed over the polypectomy site to prevent further bleeding. Histology from the initial biopsy and subsequent resection demonstrated fragments of foveolar hyperplasia and lobulated capillary hemangioma with focal granulation tissue, which is characteristic of PG (Figures 2(b)–2(d)). The capillaries were lined by single layer of cytologically bland endothelial cells. Neutrophils were frequently seen in the lesion. No* Helicobacter pylori* or other organisms were identified. Four months following PG resection, the patient's hemoglobin was stable and he denied any further episodes of melena.

**Figure 3:**
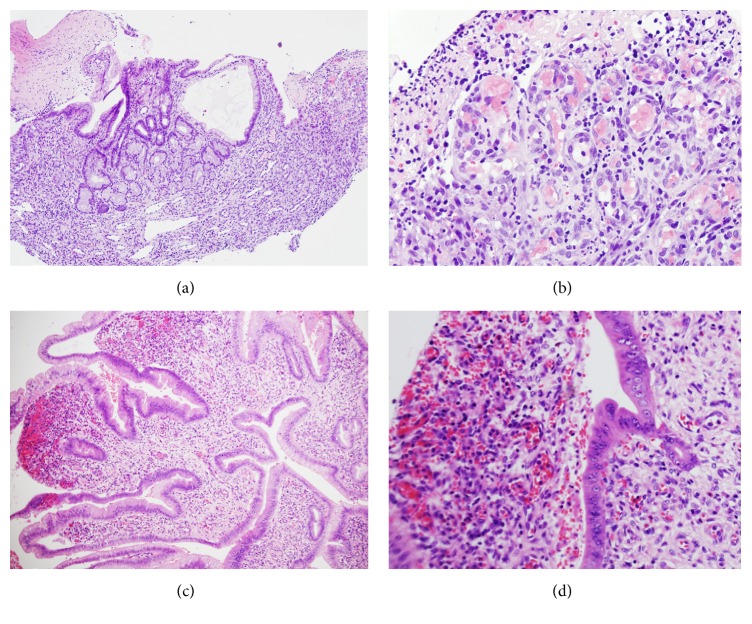
(a-b) Pyogenic granuloma of the stomach (Case 3). (c-d) Pyogenic granuloma of the ileum (Case 4). Low power views ((a) and (c), H&E, 100x) reveal gastrointestinal mucosa with variable ulceration. High power ((b) and (d), H&E, 400x) exhibits lobular arrangement of capillary proliferation and active inflammation.

*Case* *3*. A 58-year-old Caucasian female with a medical history significant for nonalcoholic steatohepatitis (NASH) cirrhosis complicated by hepatic encephalopathy, ascites, banded and eradicated esophageal varices, gastric antral vascular ectasia (GAVE) treated on multiple occasions with radiofrequency ablation, and recurrent hospitalizations for melena and anemia presented to the emergency department for three days of melena and altered mental status. Coagulation profile was normal and her hemoglobin was 9.8 g/dL, essentially unchanged from her baseline. An EGD demonstrated small esophageal varices with no stigmata of bleeding, GAVE, and a normal duodenum. Focal radiofrequency ablation of GAVE was performed in the gastric antrum using the Halo-90 radiofrequency ablation catheter. Encephalopathy resolved with lactulose and she was discharged. An outpatient surveillance EGD one month later revealed a gastric antral papule with no stigmata of recent bleeding in addition to demonstrating the small esophageal varices and GAVE. Biopsies with a cold forceps of the gastric papule induced some bleeding that needed treatment with Argon Plasma Coagulation followed by clipping. Histological examination of the gastric papule was consistent with PG (Figures 3(a)-3(b)).

*Case* *4*. A 50-year-old African American female was admitted with abdominal pain and generalized abdominal distention. Her past medical history was notable for adult polycystic liver. An exploratory laparotomy revealed a small bowel perforation. She then underwent a small bowel resection for perforated Meckel's diverticulum with primary small bowel anastomosis. Histological examination of the resected small bowel revealed an incidental finding of PG at the site of Meckel's diverticulum (Figures 3(c)-3(d)).

## 3. Discussion

PG of the gastrointestinal tract has the same histopathological features as those observed in the skin and oral mucosa, which are best described as a capillary hemangioma arranged in a lobular pattern and filled with clusters of small capillary vessels and a single layer of endothelial cells [2]. Granulation tissue and a neutrophilic infiltrate may also be present [3]. Given this, the main pathological differential diagnosis of PG includes bacillary angiomatosis (BA), Kaposi's sarcoma (KS), or inflammatory and/or hyperplastic polyps [4].

The histological pattern of BA is similar to PG because of the architectural pattern of endothelial cells, but BA can be distinguished by the Warthin-Starry stain. KS is characterized by a proliferation of blood vessels, inflammation, and spindle cells. Immunohistochemistry with antibodies against human herpesvirus-8 can be used to distinguish this lesion from PG. Inflammatory polyps may also have neutrophilic infiltrates and granulation tissue, but they lack the characteristic capillary lobular arrangement of PG. Immunohistochemistry for CD31 or CD34 can be used to identify endothelial cells and distinguish PG from inflammatory polyps.

While PG is rarely reported in the literature, the actual incidence is probably much higher. We have diagnosed four cases at our institution all within the past year (approximately 8% of all GI PG cases ever reported). Our speculation is that PG of the GI tract is a commonly unrecognized or misdiagnosed lesion. For instance, it may be incorrectly diagnosed as an inflammatory and/or hyperplastic polyp. This is problematic because that lesion is not a possible source of anemia as PG is.

The mechanism underlying PG pathogenesis is not well understood. As the name suggests, it previously was thought that PG arose from an infectious etiology. Most current theories at least partially involve PG arising from a reactive process against mucosal irritation, such as trauma. However, most patients do not report previous trauma at the site of PG [5], suggesting that another component is involved. For instance, hormonal influences could also play a role, as mucosal PG lesions are more likely to occur in pregnant women [6]. One other proposed precipitating factor is liver cirrhosis as it has been noted to be a common underlying disorder in some patients with GI PG [7, 8]. This association might be caused by retrograde dilatation of capillary blood vessels secondary to portal hypertension that causes venous stasis and leads to the growth of PG [8].

There are approximately 50 cases of GI PG in the English literature indexed on MEDLINE (Table 1). The most commonly reported anatomical locations are the esophagus [9–15], small bowel [2, 16–22], and the colon [4, 23–25]. The most common clinical manifestation is anemia; 39 (79%) patients presented with symptoms of bleeding. Initial hemoglobin values were reported for 17 patients; median hemoglobin was 8.1 g/dL (range: 4–16.6 g/dL). Other presentations are rarely reported and depend on the anatomical location of the PG. For instance, esophageal lesions can present with dysphagia [13], stomach lesions as gastritis [26], small intestine lesions as intussusception [25, 27], or rectum lesions as tenesmus [28]. Four (8%) patients were asymptomatic and their PG lesions were found incidentally.

The median age of patients at time of PG diagnosis was 59 years (range: 0.3–86 years). 71% of patients were older than 50 when diagnosed with GI PG. However, there are reports of GI PG in the pediatric population [25, 27]. Men seem to be more affected than women: 29 (60%) of the patients were men and 19 (40%) were women.

The endoscopic appearance of PG lesions is usually a smooth and ulcerated surface that appears friable. The color ranges from bluish-red to sanguine with a superficial white or opaque film covering. As with our third patient, they can grow quickly and then stabilize in size [29]. PG size was reported for 37 patients; median PG diameter was 15 mm (range: 4–33 mm). Only 8 lesions (22%) were larger than 20 mm. PG typically only involves the mucosa but can extend into the submucosa [13] and even the full thickness of the luminal wall [30].

Resection of PG lesions is necessary in patients with anemia, but postresection bleeding is a potential complication [31]. Resection is most commonly accomplished by endoscopic mucosal resection, endoscopic polypectomy, or surgical resection. Of those, endoscopic polypectomy was by far the most common treatment method. Regardless of treatment modality, recurrence is rare. Length of follow-up after PG resection was recorded for 19 patients, and only 2 (11%) of those patients had recurrence after resection [23, 28]. However, median follow-up was only 8 months (range: 1–39 months).

In conclusion, PG of the GI tract is a very rarely reported clinical entity. However, the actual incidence may in fact be higher if this lesion is commonly unrecognized or improperly diagnosed as an inflammatory or hyperplastic polyp. It is important for the clinician to properly recognize PG as a source of anemia.

## Figures and Tables

**Table 1:** Reported cases of gastrointestinal pyogenic granuloma.

Reference	Sex, age^*∗*^	Comorbidity	Presenting symptom(s)	Location	Diameter (mm)	Treatment	Follow-up (months)
Shin et al. [32]	M, 40	NA	Melena	Cecum	25	Endoscopic polypectomy	6
Nakaya et al. [33]	F, 59	Hepatic cyst	Melena	Descending colon	15	Endoscopic polypectomy	NA
Thibault et al. [34]	M, 54	NA	Abdominal pain, hematochezia	Descending colon	20	Endoscopic polypectomy	NA
Meyer-Herbon et al. [23]	M, 54	Gastric adenocarcinoma	Hematochezia	Descending colon	10	Endoscopic polypectomy	8
Park et al. [35]	F, 62	NA	Anemia	Duodenum	10	Endoscopic polypectomy	14
Hirakawa et al. [36]	M, 60	End-stage renal disease	Anemia	Duodenum	8	Endoscopic polypectomy	6
van Vilet et al. [9]	M, 65	Cardiovascular disease	Melena, anemia	Duodenum	10	Endoscopic mucosal resection	1
Meeks et al. [37]	M, 59	Hepatitis C	Melena	Duodenum	8	Endoscopic polypectomy	NA
Craig et al. [10]	M, 31	Testicular carcinoma, lymphoma	Reflex esophagitis	Esophagus	25	Endoscopic polypectomy	NA
Hoekstra et al. [11]	M, 15	Hiatal hernia, reflux esophagitis	Dysphagia, weight loss, vomiting	Esophagus	NA	Endoscopic polypectomy	NA
Okada et al. [12]	M, 56	NA	Chest pain	Esophagus	NA	Endoscopic mucosal resection	NA
Van Eeden et al. [13]	F, 55	NA	Hematemesis, dysphagia	Esophagus	11	Endoscopic polypectomy	NA
Tajika et al. [38]	M, 63	Esophageal cancer	Asymptomatic	Esophagus	15	Endoscopic polypectomy	NA
Iwamuro et al. [39]	M, 78	None	Gastritis	Esophagus	10	Biopsy only	39
Seoung et al. [14]	M, 58	NA	Asymptomatic	Esophagus	10	Endoscopic mucosal resection	6
Seoung et al. [14]	M, 54	None	Asymptomatic	Esophagus	5	Endoscopic mucosal resection	6
Hirata et al. [16]	F, 82	NA	Melena	Ileum	NA	Segmental resection	NA
Kikuchi et al. [40]	F, 86	Cardiovascular disease	Melena	Ileum	7	Segmental resection	NA
Nagoya et al. [17]	F, 63	None	Anemia	Ileum	7	Endoscopic polypectomy	NA
Stojsic et al. [27]	F, 13	NA	Intussusception	Ileum	40	Segmental resection	NA
Van Eeden et al. [13]	F, 55	NA	Melena, anemia	Ileum	9	Segmental resection	NA
Chou et al. [7]	M, 58	Hepatitis B, cirrhosis	Melena	Ileum	10	Segmental resection	6
Chou et al. [18]	F, 67	Hepatitis B	Melena	Jejunum	15	Endoscopic polypectomy	NA
Iravani et al. [19]	F, 53	Metastatic melanoma	Melena	Jejunum	16	Segmental resection	NA
Misawa et al. [20]	M, 58	NA	Anemia	Jejunum	19	Segmental resection	NA
Moffatt et al. [2]	M, 78	NA	Anemia	Jejunum	20	Segmental resection	NA
Katsurahara et al. [41]	M, 65	History of stroke	Melena	Jejunum	33	Segmental resection	18
Shirakawa et al. [42]	M, 72	NA	Melena	Jejunum	NA	Endoscopic polypectomy	13
Kuga et al. [21]	M, 55	None	Anemia	Jejunum	4	Endoscopic polypectomy	10
Field et al. [24]	F, 80	Atrial fibrillation	Hematochezia	Rectum	NA	Endoscopic polypectomy	NA
Giaccaglia et al. [28]	M, 43	Hypertension, gout	Hematochezia, tenesmus	Rectum	30	Endoscopic polypectomy	NA
Moparty et al. [43]	F, 26	NA	Asymptomatic	Rectum	5	Endoscopic polypectomy	NA
Blanchard et al. [44]	F, 5	None	Hematochezia	Rectum	4	Endoscopic polypectomy	NA
Castle et al. [45]	M, 16	None	Hematochezia	Rectum	NA	Transanal mucosal sleeve resection	24
Hamada et al. [46]	M, 59	Chronic myelogenous leukemia	Altered bowel habit	Rectum	10	Endoscopic polypectomy	NA
Garofalo et al. [25]	M, 0.3	NA	Intussusception	Sigmoid colon	20	Segmental resection	NA
González-Vela et al. [4]	F, 62	NA	Hematochezia	Sigmoid colon	20	Endoscopic polypectomy	8
Chen et al. [47]	M, 36	None	Melena, weight loss	Sigmoid colon	Range: 4–8^*∗*^	Endoscopic polypectomy	1
Yamashita et al. [22]	M, 61	Diabetic nephropathy	Melena	Small bowel	15	Segmental resection	NA
Quiros et al. [3]	F, 67	None	Anemia	Stomach	10	Endoscopic polypectomy	NA
Erarslan et al. [48]	M, 64	None	Hematemesis, melena	Stomach	8	Endoscopic polypectomy	NA
Malhotra et al. [31]	F, 40	None	Anemia	Stomach	NA	Endoscopic polypectomy	NA
Val-Bernal et al. [49]	F, 72	Hypothyroidism	Melena	Stomach	25	Endoscopic polypectomy	NA
Val-Bernal et al. [49]	M, 66	Cardiovascular disease	Anemia	Stomach	7	Endoscopic polypectomy	NA
Kusakabe et al. [50]	M, 82	NA	Melena, anemia	Stomach	30	Endoscopic polypectomy	12
Shibata et al. [26]	M, 35	H. pylori infection	Pain	Stomach	20	Endoscopic polypectomy	2
Hocke and Bosseckert [51]	F, 60	Liver cirrhosis, colon cancer	Anemia	Transverse colon	NA	Hemicolectomy	NA
Blanchard et al. [44]	F, 2	Liver transport for biliary atresia	Fever, hematochezia	Transverse colon	NA	Endoscopic polypectomy	NA
Yan et al. [52]	M, 40	None	Hematochezia	Transverse colon	25	Endoscopic polypectomy	3

^*∗*^Age in years.
